# Characteristics of Ions Emission from Ultrashort Laser Produced Plasma

**DOI:** 10.1038/srep38256

**Published:** 2016-12-01

**Authors:** Ahmed M. Elsied, Nicholas C. Termini, Prasoon K. Diwakar, Ahmed Hassanein

**Affiliations:** 1 Center for Materials under Extreme Environment (CMUXE), School of Nuclear Engineering, Purdue University, West Lafayette, IN 47907, USA.

## Abstract

The dynamic characteristics of the ions emitted from ultrashort laser interaction with materials were studied. A series of successive experiments were conducted for six different elements (C, Al, Cu, Mo, Gd, and W) using 40 fs, 800 nm Ti: Sapphire laser. Time-of-flight (TOF) ion profile was analyzed and charge emission dependencies were investigated. The effects of incident laser interaction with each element were studied over a wide range of laser fluences (0.8 J/cm^2^ to 24 J/cm^2^) corresponding to laser intensities (2.0 × 10^13^ W/cm^2^ to 6.0 × 10^14^ W/cm^2^). The dependencies of the angular resolved ion flux and energy were also investigated. The TOF ion profile exhibits two peaks corresponding to a fast and a slow ion regime. The slow ions emission was the result of thermal vaporization while fast ions emission was due to time dependent ambipolar electric field. A theoretical model is proposed to predict the total ion flux emitted during femtosecond laser interaction that depends on laser parameters, material properties, and plume hydrodynamics. Incident laser fluence directly impacts average charge state and in turn affects the ion flux. Slow ions velocity exhibited different behavior from fast ions velocity. The fast ions energy and flux were found to be more collimated.

Dynamics of ions emission are important to study for a wide spectrum of applications either medical^1^, industrial or academic, such as pulsed laser deposition^2^, laser induced breakdown spectroscopy^3^, laser assisted mass spectrometry^4^, ion implantation^5^, and light source generation^6^. With nanosecond pulsed lasers, the ablation of metals is accompanied by large-heat affected zones, as well as the formation and ejection of molten material^7^,^8^. On the other hand, femtosecond lasers offer much different features that include very high power deposition, reduced presence of splashed molten material, and negligible heat-affected zones^9^. So collateral damage due to shock waves and heat conduction is negligible in case of femtosecond laser material ablation. Since the thermal conduction into the target during the laser pulse duration can be neglected, the ultrashort laser pulse ablation process during the pulse duration is considered to be direct solid-plasma transition. This process allows heating the lattice in picoseconds forming plasma. This has the advantage of much higher precision and quality in the ablation and machining of metals^7^. Therefore, it is important to investigate in more details the physics of femtosecond laser interaction with metals and dielectrics, due to their desire in many applications.

To gain more understanding, several experiments were conducted to analyze the ablated plasma plumes^10^,^11^,^12^ using ultrashort lasers at fluences close to ablation threshold fluences. These experiments provided insight into how the plume itself is formed and how its components behave. Analysis of the plume shows that neutral atoms fly behind the charged particles. Further investigations on the charged particles at higher laser fluences provided more details about their nature and their behavior. Some of these studies^13^,^14^,^15^ focused only on the ions revealed that two types of ions based on their energy are emitted after laser irradiation, slow and fast ions. Slow ions were shown to follow shifted Maxwell Boltzmann (SMB) distribution and fast ions follow Gaussian distribution^15^. This further confirmed that slow ions emission is due to thermal vaporization; sometimes referred to these ions as thermal ions. The fast ion or non-thermal ion emission mechanism is different, and is still under debate. In addition, the nature of these fast ions and their dependence on laser and target material parameters still needed to be explored, and these are the topic of this article.

In these experiments, we studied the TOF ion emission spectra for different elements of varying atomic mass (C, Al, Cu, Mo, Gd, and W) at different fluences starting from near ablation threshold fluence to relatively high fluences. We also studied the angular distribution of the ion flux and kinetic energy for each element. We then analyzed the TOF spectra to further explain the nature and emission mechanisms of non-thermal and thermal ions during ultrafast laser interaction and how they depend on both laser and material properties. Preliminary modeling was provided for better understanding and explanation of these experimental results.

## Experimental Setup

**Figure 1:**
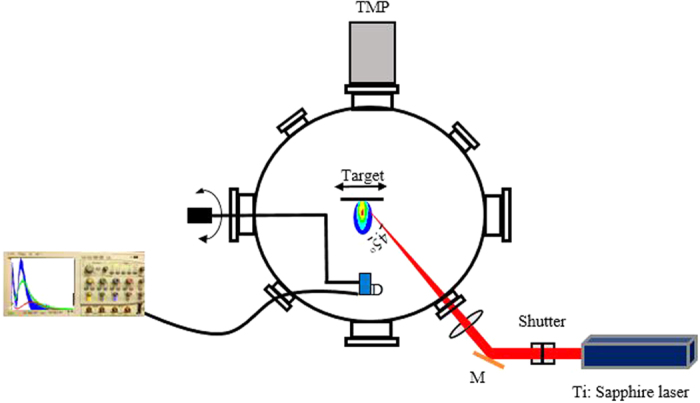
Schematic diagram of the experimental setup. As shown the laser beam has a 45° incident angle. Focused inside the chamber through 40 cm lens and the beam goes into the vacuum chamber through a quartz window. Ion collector is mounted on angle manipulator holder and kept at 9.2 cm away from the target surface.

A schematic diagram of the experimental setup is illustrated in Fig. 1. The laser source is a Ti: Sapphire system consisting of an oscillator and a chirped pulse amplifier. The oscillator pulse with 40 fs and 800 nm wavelength goes through a stretcher, a regenerative amplifier, and eventually is compressed to an output P-polarized laser pulse of maximum laser energy of 7.5 mJ, and 40 fs pulse duration at full width at half maximum. Laser output energy is tunable by using a set of half waveplate, and a thin film polarizer positioned before the compressor. Pure targets of 99.99% (C, Al, Cu, Mo, Gd, W) and detector (Faraday cup ion collector (IC)) are placed under vacuum (10^−6^ Torr) in a stainless steel vacuum chamber. The femtosecond pulses are focused onto the targets with 45° incidence angle via 40 cm plano-convex lens that yield elliptical spot with size of 3 × 10^−4^ cm^2^. The targets are placed on a remotely controlled XY translation stage to have a fresh surface and to move from one target to another.

The experimental setup allows the measurements of the ion emission dynamics by measuring the ions time-of-flight (TOF) using faraday cup ion collector (IC) (Kimball Physics, Inc., model FC-71A). The IC is mounted on an angle manipulator allowing changing the measurement angle from −π/2 to π/2 with respect to normal to the target surface to measure the angular resolved ion flux and velocity. It is kept at 9 cm away from the target surface and negatively biased at −40 V to repel all the electrons and collect only the ions. The IC has a front aperture with 5 mm diameter hole. A shutter is placed in front of the laser beam to allow only single shot. This shutter is controlled by a time delay generator, which receives an advance signal from the laser system and triggers the shutter. The output signals from FC are acquired across 50-Ω load resistor using 1 GHz oscilloscope (Tektronix TDS5104). A fast photodiode was used to trigger the oscilloscope simultaneously with the laser pulse to record the ion signals. Few laser shots were used initially to clean the target surface at first. The number of cleaning laser shots varied from target to target, so the criteria was to keep the cleaning laser shots until the signal becomes very stable. The ion signals reported below are obtained after cleaning and averaged over 10 successive laser shots for good statistics. Refreshing the target used to avoid drilling the targets as well. The laser intensity used in this work varied from 2 × 10^13^ W/cm^2^ to 6 × 10^14^ W/cm^2^ that corresponds to laser fluence of 0.8 J/cm^2^ to 24 J/cm^2^. The laser beam contrast ratio is 1.0 × 10^−6^ so no pre-pulse effects on these results.

## Experimental Results

In the following, we will discuss the various experimental results of this work. The TOF ion flux profile for different metals will be discussed first. Second, the effect of incident laser fluence on ion flux. Third, the effect of the incident laser fluence on ion velocity. Eventually, the angular resolved ion flux and kinetic energy.

### TOF ion flux profile

**Figure 2:**
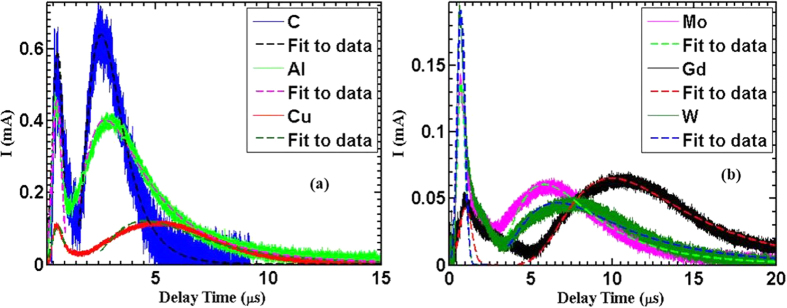
(**a**,**b**) Ion flux TOF signal measured at 0° IC angle with respect to normal to target surface, and at 9 cm away from the target surface. 1.2 J/cm^2^ (2.9E13W/cm^2^) laser fluence was used with 45° laser incidence angle.

Figure 2(a,b) shows the ion TOF signal for C, Al, Cu, Mo, Gd, and W. By analyzing the ion current in Fig. 2, several observations and trends can be concluded. The first important observation is that all the elements have clear and resolved double peaks. The first peak is a fast peak, which is predicted to be caused by space charge separation^15^,^16^,^17^,^18^. However, several other mechanisms have been proposed to also cause fast ion generation. One mechanism proposed is that, the fast peak is due to light contaminants on the surface of the material^19^,^20^. However, this can be neglected in our experiments since several cleaning laser shots were taken before data was acquired. The cleaning shots were acquired till the signal gets stable, eventually the recorded signal is a result of 10 successive laser shots. Another mechanism is that the rising edge of the laser creates a vapor, which is heated by the back end of the laser producing hot electrons due to charge separation mechanism^13^. However, this can also not be the case since the laser pulse in our experiment (40 fs) terminates way before the energy can be transferred from electrons into the lattice to produce a vapor, this process takes several picoseconds. Consequently, the most probable fast ion emission mechanism as proposed by ref. 18 after comprehensive study, was the space charge mechanism. In this phenomenon, a fraction of the electrons population, undergoes negligible energy-changing collisions are emitted leaving ions behind them due to their inertia. This forms ambipolar electric field. As a result, hot ions are ejected and accelerated to high energies, as also proposed by refs 15, 16, 17. The validity of this physical explanation will be discussed later. The second peak is a thermal peak, which is caused by thermal vaporization of the material after energy is transferred into the lattice by electrons.

To verify the thermal and non-thermal nature of the slow and fast peaks, fitting was applied to each peak. It was found that the fast peak was best fitted to a Gaussian distribution which is represented by equation 1. The slow peak was best fitted to a shifted Maxwellian distribution (SMB), represented by equation 2.


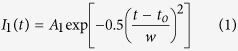



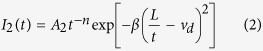


The Gaussian peak’s parameters are simple with *t*_*o*_ being the peak center, and *w* being the peak width and A_1_ being the amplitude. The SMB distribution is less obvious and has several important properties pertaining to the plasma. The plasma ion temperature is represented by *β* = *M*/2*k*_*B*_*T*_*i*_ which is determined by the fluence. The variable *n* is indicative of plasma density with higher *n* values corresponding to higher plasma density. *L* is the distance from the target to the IC, was kept at 9.2 cm. Both *v*_*d*_ and *A*_*2*_ are fitting parameters, with *v*_*d*_ controlling the peak shift. From the fitting it was found that, *w* tends to increase with increasing target atomic mass at the same laser fluence. And *T*_*i*_ was found to vary from 0.5 to 12 eV, and follow the same trend as slow ion velocity. In the following sections the trend of *T*_*i*_ that represents the slow ion energy will be discussed in details.

Several clear trends can be generally determined from these signals, most particularly with varying atomic mass. The first obvious trend is that the peak intensity of each signal for both the fast and thermal peaks decreases generally with increasing atomic weight. This trend is very clear for the lighter elements, with carbon having the highest fast and thermal peaks, with Al and Cu following behind. For the heavier elements, the fast peaks follow this trend clearly as well. The thermal peaks however are less clear with W having the highest peak intensity, and Gd and Mo having nearly the same peak intensity.

**Figure 3:**
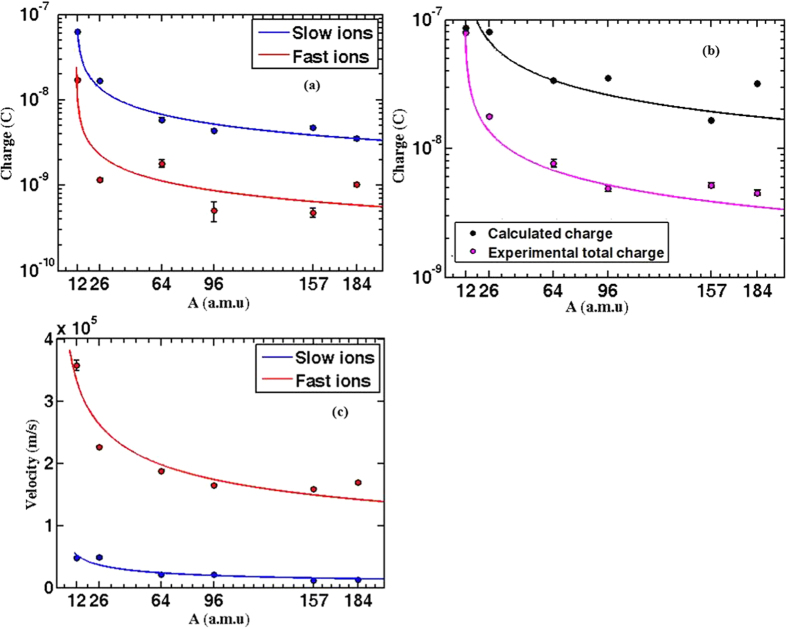
(**a**) Shows the emitted charges dependence on the atomic weight for different elements for both fast and slow ions. (**b**) Shows the calculated total charges using equation 5 and the experimental total emitted charge, data were fitted as A^−0.75^ based on equation 5. (**c**) Indicates how the velocity changes with ion mass. Best fitting shows that Velocity scales as M^−0.5^, for slow and fast ions. Data were measured at 0° IC angle and 5 J/cm^2^ (1.25E14W/cm^2^).

The other important trend that can be observed from these peaks is the TOF or velocity of the thermal ions component. As expected from first principles, velocity is inversely proportional to the molecular mass of the material for the same energy deposited. This can be clearly seen from the signals of the thermal peaks, with carbon ions arriving the quickest and tungsten ions arriving the slowest. It is not clear from these signals that the fast peaks change significantly, but that is due to the fact that the changes are on the order of nanoseconds, and the change can be clearly seen in Fig. 3c.

Another observation is the expansion time of the ablated plume. One can clearly see that as atomic weight increases, plume expansion time increases. This is deduced by the expanding peak widths of the thermal peaks. This trend is clearly represented in Fig. 2.

Figure 3 shows a comparison of ion charges and velocities for different elements. This is the result of integration over the fast peak and the thermal peak regions separately of the ion TOF divided by 50-Ω resistance (Eq. 3).


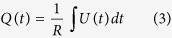


*U(t), R,* and *Q* are the time dependent ion signal in voltage measured by IC, 50-Ω load resistor and total charge collected by IC, correspondingly. It is evident from Fig. 3(a) that the ejected charges tend to decrease with increasing target atomic weight for the same laser intensity. The highest charge yield results from laser-carbon interaction, which is one order of magnitude higher than the lowest charge yield results from laser-gadolinium interaction. It is also noticed from Fig. 3(a) that the total flux is dominated by the thermal ions. The maximum ion charge, *Q*_*max*_, that can be ejected from a target can be estimated by assuming that all atoms, *n*_*i*_, lie in a volume defined by laser spot size on target, *S*, and depth defined by optical skin depth, *l*_*s*_, given by 
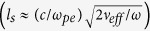
21 where *ω*_*pe*_, ω, and *v*_*eff*_ are electron plasma frequency, laser frequency and effective collision frequency, respectively, will be ionized. So *Q*_*max*_ can be expressed as 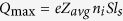
22. The ion emission was found to follow certain angular distribution given by equation 4^23^,^24^.


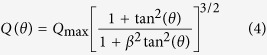


where *β* is the forward peaking factor, which represents the ratio between longitudinal and transverse axis of the emitted plume. This factor varies from 2 to 5 based on the target material, and will be discussed in more detail later. Equation 4 specifies the fraction of the charges that can be detected in terms of both the geometry of the ion detector and the material properties of the target. The distance between the detector (IC) and surface target has an effect on collected ion charges. This effect is mainly due to electron-ion recombination and ion flux attenuation as it travels toward the detector. The first can be neglected since the experiment was carried out under vacuum (10^−6^ Torr), and the second can be scaled as *1/r*^*2*^, where *r* is the distance between the ion detector (IC) and target surface. The final expression for the total ion charges collected by the detector (IC) in terms of plasma plume geometry, laser parameters and material properties is given by equation 5.


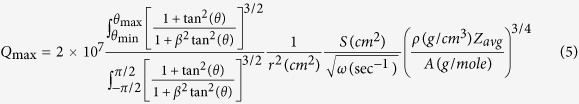


The average ionic charge state, *Z*_*avg*_, can be calculated as a function of electron temperature by solving Saha equation^25^ and consider the nonideal effect of plasma^26^, the detailed solution of Saha equation can be found in reference^27^. Hence the electron temperature can be correlated to incident laser intensity through equation 6^28^,^29^.


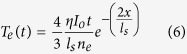


The emitted charges are calculated for each element (black dots in Fig. 3(b)) and scaled as A^−0.75^ (black solid line in insertion as it is proposed by Eq. 5). The theoretical flux qualitatively is in good agreement with the experimental data as it exhibits similar trend, while quantitatively it is higher, as shown in Fig. 3(b). One reason for that is due to the uncertainty in calculating the laser penetration depth where the maximum value of the collisional frequency is used (*v*_*ei*_ ≈ ω_*pe*_)^21^,^30^,^31^, instead of correcting for the collision frequency decrease with increasing electron temperature^30^ (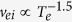). Moreover, we assumed that the incident laser energy is uniformly distributed, thus all the atoms inside the volume (*Sl*_*s*_) will be ionized. However, in practice neutral atoms form in addition to charged particles.

In Fig. 3(c), the velocities of both fast and slow ions are plotted versus atomic mass. For the two ion species, the velocity decreases with increasing the mass, as should be expected (*V* ∝ *M*^−0.5^) assuming the same amount of laser energy is absorbed in each material. The highest velocity is shown for carbon, the lightest element, to be ~4 × 10^5^ m/s and 5 × 10^4^ m/s for fast and slow ions, respectively. Another observation is that the velocity of fast species is almost an order of magnitude higher than that of the slow species. The hot electron temperature, *T*_*h*_, that induces those fast ions can be estimated from their corresponding hot ion velocities by assuming that the acceleration scheme looks like a capacitor consists of two plates of opposite charges. Consequently, a time dependent ambipolar electric field forms between these two plates. This field pulls out ions from the target surface only if the ions gain energy larger than the binding energy (heat of vaporization) of ions. Accordingly, the ions will gain energy as they follow the electrons and this will then decelerate the electrons^15^. Thus the acceleration process will be slowed down or truncated, as presented in many studies^32^,^33^,^34^,^35^. Once the ions catch up with the electrons, they continue to drift with constant velocity, i.e., no more energy can be gained. Based on the model discussed by Gibbon^32^, the ions maximum kinetic energy can be estimated using the following equation:


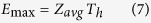


Consequently, the hot electrons temperatures can be estimated from this model by knowing the fast ions kinetic energy, which varies from 0.5 keV for C to 10 keV for W. Similarly, from thermal electron temperature equation 6, the cold electron temperature that induces thermal ions can be calculated, and it ranges from 4.2 eV for Al to 11 eV for Gd.

### Effect of laser fluence on emitted ion flux

**Figure 4:**
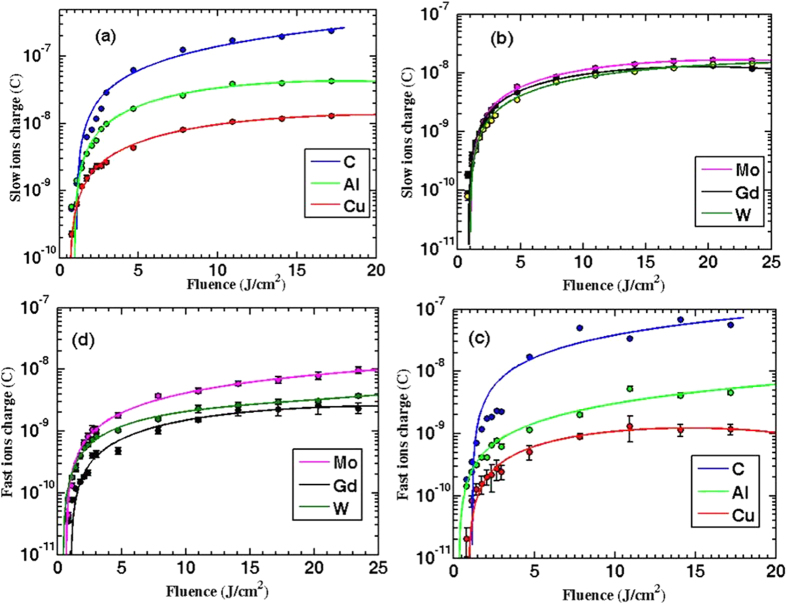
Illustrates a linear increase for both fast and slow ion charges. This occurs through two regimes. First regime shows Rapid increase rate at low fluence, followed by second regime in which slow increase rate is observed as the laser fluence increases. Data presented here are measured at 0° and 9 cm away from the target.

**Figure 5:**
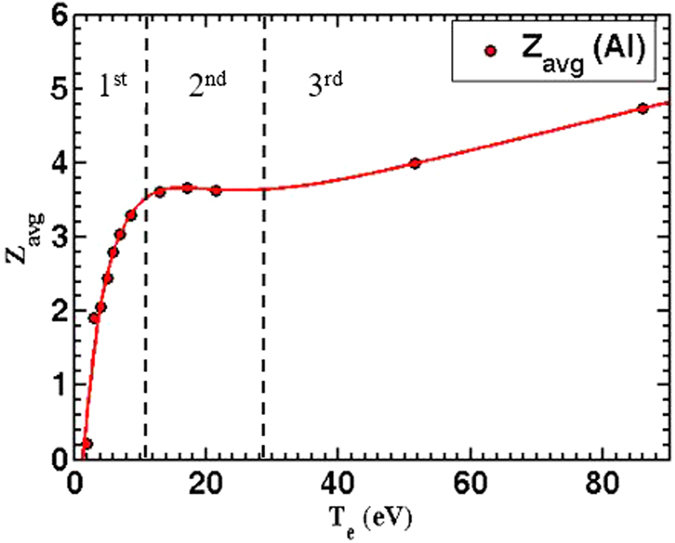
Solution of Saha equation^25^ for Aluminum at STP results in Al average charge state for different electron temperature. Three regimes are shown. 1^st^ regime at low temperature, the increase rate of average charge state is high. 2^nd^ regime average charge state levels off over wide range of electron temperature. 3^rd^ regime in which the average charge state started to increase again.

In the previous section, it was shown that the ion flux depends on the laser, material, and hydrodynamics properties. More details were illustrated using equation 5. Figure 4 shows how the ion flux changes for each element as function of the incident laser fluence. All the six elements exhibit a very consistent trend by showing two regimes. The first regime at a low laser fluence (<*4 J/cm*^2^) is characterized by a rapid rising rate in the ion flux. This is followed by a second regime at a higher laser fluence (>*4 J/cm*^2^), described by a slow ion flux increase rate. The trend in the ion flux is in a good agreement with previous experiements^21^,^22^. It should be emphasized that the IC signal is sensitive to the charge not to the number of ions. In other words, IC doesn’t differentiate between two singly ionized ions or one doubly ionized ion; both will yield the same count. So the impact of laser fluence on detected ion charges can be explained as follow: In the first regime, increasing laser irradiation tends to increase the electron temperature thus it induces ions with higher ionization state. Figure 5 shows theoretically calculated Al ions average charge state as an example. This laser fluence impact on average charge state, hence the detected ion charges, is evident by comparing the 1^st^ region in Fig. 5 (*T*_*e*_ < *11* *eV*) with the 1^st^ region in Fig. 4 (*fluence* < *4 J/cm*^2^), where both show a rapid increase in average charge state and flux, respectively. Also, experimentally ions with higher ionization states were detected and measured^13^,^36^. Moreover, the effect of ions ionization state is investigated here theoretically by equation 5. In the second region that corresponds to laser fluence higher than *4* *J/cm*^2^ and an electron temperature that lies between *11 eV* and *30 eV*, the ionization state of the emitted ions is independent on the incident laser fluence (see 2^nd^ region in Fig. 5), thus the slight increase in the detected ion charge in 2^nd^ region is due to increasing in number of ions rather than altering the ionization state of the ions. Based on this observation, a new regime corresponding to the 3^rd^ region in Fig. 5, can be expected at laser fluence higher than 40 J/cm^2^ or intensities higher than 1.0 × 10^15^ W/cm^2^ calculated from equation 6.

### Effect of laser fluence on emitted ion velocity

**Figure 6:**
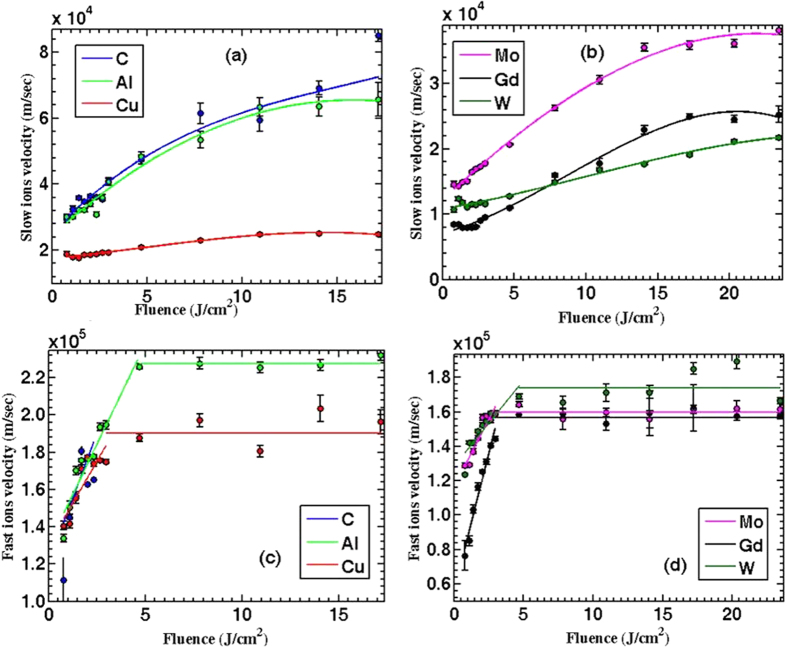
The changes in ion velocity due to increasing laser fluence. Slow ions shown in (**a**,**b**) have different trend from that shown by fast ions (**c,d**). Data presented here are measured at 0° and 9 cm away from the target.

**Figure 7:**
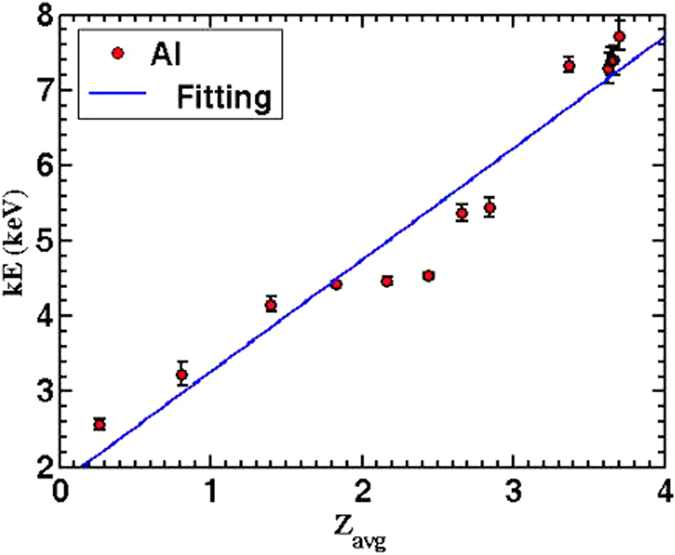
Illustrates the dependence of the fast aluminum ions kinetic energy on the ions average charge state calculated by solving Saha equation^25^. Fluctuation of laser energy from shot to shot causes some point to deviate at intermediate Z_avg_.

Figure 6 illustrates the change in the ion velocities as function of the incident laser fluence. Slow ion velocity is one order of magnitude less than fast ion velocity. Slow ions exhibit a sublinear increase with increasing incident laser fluence in a good agreement with previous results^15^,^24^. Fast ion velocity shows linear increase with laser fluence less than 4 J/cm^2^, then it levels off at higher laser fluences, >4 J/cm^2^. Two points are worth noticing here: (1) Comparing Figs 6(a,b) with 6(c,d), different trends in the ion velocity indicate that the acceleration mechanisms are clearly different as discussed earlier, (2) comparing Fig. 6(c,d) with Fig. 5, reveals that fast ions kinetic energy is linearly proportional to ionic charge state, see Fig. 7. The last observation reveals that the fast ion emission mechanism is due to space charge separation in which the ions’ kinetic energy is linearly proportional to ionic charge state given by equation 7. The ions’ kinetic energies can be scaled on the basis of the mechanism originating them in terms of electron temperature whether thermal or non-thermal (*T*), work function (*φ)* and heat of vaporization (*Ω*) as *kE*_*i*_ = (*T* − *ϕ* − Ω). For slow ions, the acceleration process occurs as a result of thermal vaporization. So as an example, Al has work function and heat of vaporization of about 4, 3 eV, respectively. This yields kinetic energy in the order of tens of eV which means an ion velocity in the order of ~10^4^ m/s which agrees with the experimental results shown in Fig. 7a.

**Figure 8:**
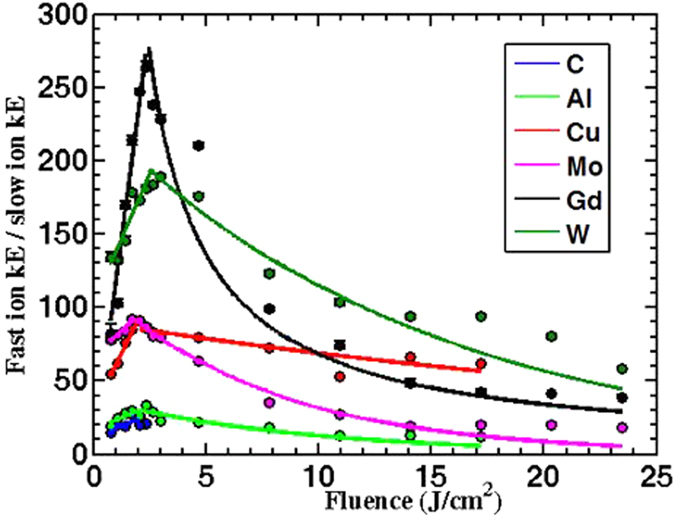
Shows that the ration between fast and slow ions kinetic energies as a function of incident laser fluence. Data presented here were measured at 0° and 9 cm away from the target.

Results presented in Fig. 8 show the difference between the two mechanisms that are responsible for ion emission, namely space charge separation for fast ions, and thermal heating for the slow ions. The relative contribution of space charge separation mechanism becomes significant at intermediate laser fluences ~ 4 J/cm^2^ (10^14^ W/cm^2^) while less significant at higher laser fluence. At lower fluences, space charge separation effect increases with increasing laser fluence. This can be explained from Fig. 6 that shows fast ions velocities level off at fluences higher than 4 J/cm^2^ (10^14^ W/cm^2^), while slow ions velocities continue to increase. At higher intensities, it is possible that both peaks overlap and becomes hard to resolve the two peaks, unless higher ionization state is reached so fast ions velocities can increase after this level off as explained in the previous section. It is noticed that the ratio is small for light elements, and increases as the ion’s mass increases. This could explain the difficulties encountered in previous work^13^,^15^ where it was hard to resolve the fast ion peak for both C and Al, and why in the case of Cu it was more noticeable.

### Angular distribution of both ion velocity and flux

**Figure 9:**
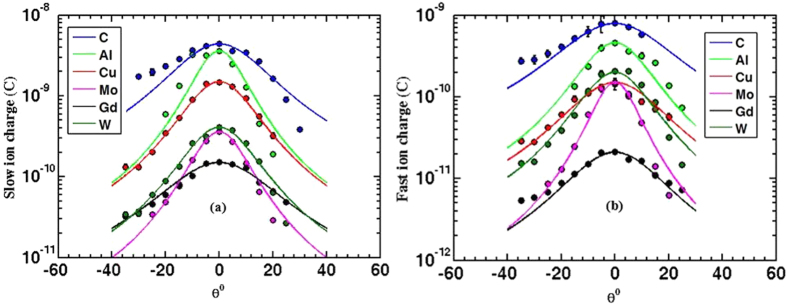
Shows the angular resolved ion flux for both fast and slow ions. Fast ion flux (**a**) seems to have narrower distribution compared to slow ion flux (**b**). Data presented here are results of 1.84 J/cm^2^ or 4.6 × 10^13^ W/cm^2^ laser target interaction with 45° incident angle, and the ions are collected by IC, 9 cm away from the target.

The angular resolved ion flux shown in Fig. 9 illustrates that the maximum ion flux peaks at an angle normal to the target surface. It can be concluded from previous published work in addition to this work that the emission direction of the ablated plasma plume is independent of the laser incident angle, ablated target, and the ion emission mechanism as well as laser pulse duration. The emission is always normal to the target surface. In contrast, with longer laser pulses, the fast ion emission peaks at larger angles^37^. The experimental data in Fig. 8 are fitted by a model given by equation 4. This model assumes isentropic and adiabatic self-similar expansion of the ablated plume at the end of laser pulse^23^.

Fitting the experimental data provides geometrical information about the ablated plume through the term *β* as a fitting parameter. This parameter measures the asymptotic ratio between the longitudinal and the transverse axis of the plume. In other words, if *β* > *1*, this means the plume is forward peaked and has an elliptical shape. This parameter was found to be larger for the case of fast ions than slow ions. The plume width, which is function of *β*, was found to be correlated to the ion yield as it decreases with increasing the ion yield^24^. In general, best fitting to the experimental data shows that *β* varies from 2 to 5.

**Figure 10:**
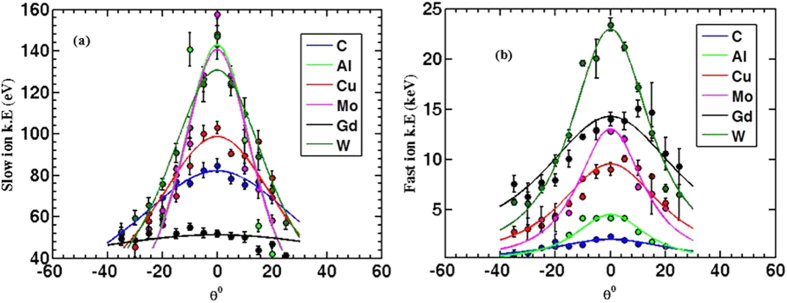
Shows the ion energy distribution flux for both fast and slow ions. Fast ions (**b**) seem to have broader distribution than slow ions (**a**). Data presented here are results of 1.84 J/cm^2^ or 4.6 × 10^13^ W/cm^2^ laser target interaction with 45° incident angle, and the ions are collected by IC, 9 cm away from the target.

Similarly, the angular distribution of ions’ kinetic energy was also studied for both fast and slow ions, see Fig. 9. Fast ions tend to be more collimated as they show narrower angular distribution than slow ions. Furthermore, as illustrated from Fig. 10 the hottest part of the plume is the plume front since kinetic energies peak at angle normal to the target surface. This is consistent with the flux angular distribution since the maximum flux is concentrated in the hottest part of the plume at the plume front.

## Conclusion

The dynamics of ultrafast laser produced ions were studied. Six elements were used to characterize the behavior of the emitted ions flux and velocity. Details of the time-of-flight (TOF) ion profile were investigated. Ambipolar electric field or space charge separation causes the observed fast ion peaks. However, the thermal ions peak is induced by thermal vaporization. Incident laser fluence, as well as material properties, control both ion flux and velocity. Different interaction regimes were found based on electron temperature, i.e., through laser fluence. In the first regime (<4 J/cm^2^) at lower temperatures less than the fermi temperature (~11 eV), the average charge-state and the ion flux are characterized by rapid increase. In the second regime at intermediate temperatures, the average charge-state levels off at value close to the number of electrons in the outermost shell, but the ion flux increases slightly due to slight increase in number of ions pulled out from the skin layer. A third regime (>40 J/cm^2^) is predicted by the average charge state behavior, and is worth studying in future. Within this regime the average charge state started to increase again but with slower rate compared to the first regime, therefore an increase in ions flux should be predicted as well. The dependence of the emitted ion flux on laser, material properties, and plume geometry were investigated experimentally and theoretically. Slow ions velocity showed sublinear increase with laser intensity, while fast ions showed a different trend similar to the average charge state behavior. The dependence of the fast ion velocity on the hot electron temperature was explained through an analytical model. This model was also used to estimate hot electron temperature. Space charge separation was found to be more significant than thermal vaporization at intermediate laser intensities and for heavier ion mass. Ion flux and energy angular distributions were measured. Energy distribution showed that hot ions are concentrated at the peak front, which indicated that ion flux should also be concentrated at the front of the peak. Fast ions flux has narrower distribution compared to slow ions flux. Fast ions were also more collimated than slow ions.

## Methods

Ultrashort laser pulses of 800 nm were used to irradiate six different solid targets with wide range of laser fluences in order to understand the ultrashort laser-material interactions including the dynamics and mechanisms of the emitted ions. Faraday cup ion collector was used to detect ions and provide ion time of flight (TOF) measurements for different materials under different laser fluences. These measurements along with theoretical models^27^,^28^,^29^ were used to estimate average ionic charge state of the emitted ions as a function of electron temperature^27^ and correlate it to incident laser intensity^28^,^29^. Furthermore, the TOF ion profile was fitted by Gaussian distribution function and shifted Maxell Boltzmann distribution function^15^ for fast and slow ions, relatively. The dependence of the maximum ion kinetic energy of fast ions on ionic charge state and hot electron temperature^32^ is used to verify that fast ions are mainly driven by ambipolar electric field or space charge separation mechanism.

## Additional Information

**How to cite this article**: Elsied, A. M. *et al*. Characteristics of Ions Emission from Ultrashort Laser Produced Plasma. *Sci. Rep.* **6**, 38256; doi: 10.1038/srep38256 (2016).

**Publisher’s note:** Springer Nature remains neutral with regard to jurisdictional claims in published maps and institutional affiliations.
